# Assessing Determinants of Programmatic Performance of Community Management of Malaria, Pneumonia, and Diarrhea in Children in Africa: Protocol and Data Collection for a Mixed Methods Evaluation of Integrated Community Case Management

**DOI:** 10.2196/33076

**Published:** 2022-03-14

**Authors:** Aliya Karim, Don de Savigny, Jean Serge Ngaima, Daniel Mäusezahl, Daniel Cobos Muñoz, Antoinette Tshefu

**Affiliations:** 1 University of Basel Basel Switzerland; 2 Swiss Tropical and Public Health Institute University of Basel Basel Switzerland; 3 Université de Kinshasa Kinshasa the Democratic Republic of the Congo

**Keywords:** iCCM, integrated community case management, systems thinking, malaria, study design, systems methods, child health, program evaluation

## Abstract

**Background:**

Integrated community case management (iCCM) is a child health program designed to provide integrated community-based care for children with pneumonia, malaria, or diarrhea in hard-to-reach areas of low- and middle-income countries. The foundation of the intervention is service delivery by community health workers (CHWs) who depend on reliable provision of drugs and supplies, consistent supervision, comprehensive training, and community acceptance and participation to perform optimally. The effectiveness of the program may also depend on a number of other elements, including an enabling policy environment, financing mechanisms from the national to the local level, data transmission systems, and appropriate monitoring and evaluation. The extent to which these factors act upon each other to influence the effectiveness and viability of iCCM is both variable and challenging to assess, especially across different implementation contexts.

**Objective:**

In this paper, we describe a mixed methods systems-based study protocol to assess the programmatic components of iCCM that are associated with intervention effectiveness and report preliminary results of data collection.

**Methods:**

This protocol uses a mixed qualitative and quantitative study design based on a systems thinking approach within four iCCM programs in Malawi, Democratic Republic of the Congo, and Niger State and Abia State in Nigeria. Routine monitoring data are collected to determine intervention effectiveness, namely testing, treatment, and referral outcomes. Surveys with CHWs, supervisors, and caregivers are performed to collect quantitative data on their demographics, activities, and experiences within the program and how these relate to the areas of intervention effectiveness. Focus group discussions are conducted with these stakeholders as well as local traditional leaders to contextualize these data. Key informant interviews are undertaken with national- and district-level program stakeholders and officers knowledgeable in critical program processes.

**Results:**

We performed 3836 surveys and 45 focus group discussions of 379 participants with CHWs, supervisors, caregivers, and traditional leaders, as well as 120 key informant interviews with district- and national-level program managers, health officers, and ministry officials. Policy and program documents were additionally collected for review.

**Conclusions:**

We expect that evidence from this study will inform child health programs and practice in low- and middle-income settings as well as future policy development within the iCCM intervention.

**International Registered Report Identifier (IRRID):**

DERR1-10.2196/33076

## Introduction

### Background

**Figure 1:**
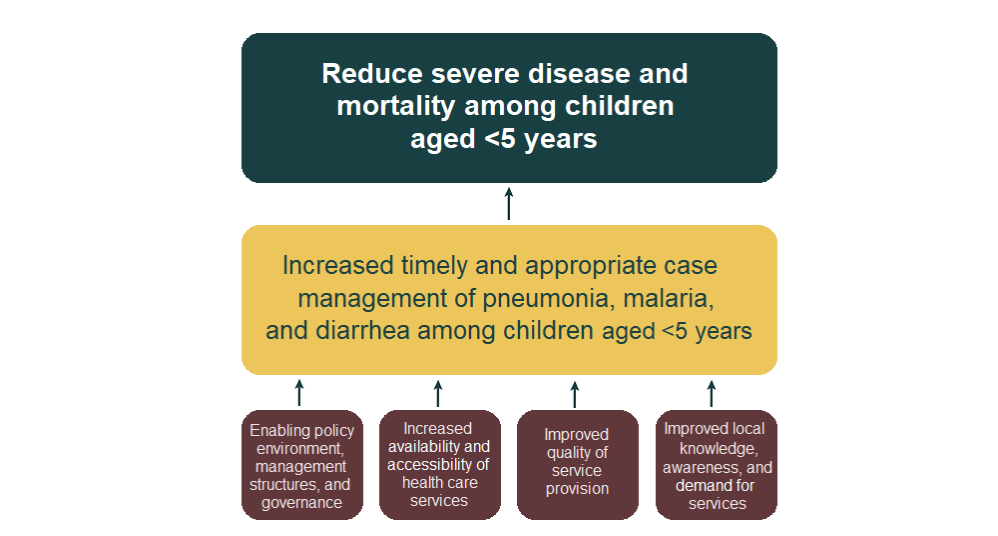
Logical framework of integrated community case management.

Integrated community case management (iCCM) is a child health strategy that aims to provide equitable access to care for children with pneumonia, malaria, and diarrhea in some of the world’s poorest and low-access areas [1]. It is focused on a community-centered model of service delivery, where trained community health workers (CHWs) provide diagnostics and treatment to children aged <5 years in assigned catchment areas. iCCM has become recognized in the global public health sphere as a critical approach to reducing child mortality, reinforced by the World Health Organization and United Nations Children’s Fund in a joint statement published in 2012 [2]. Figure 1 provides a logical framework of iCCM, its pillars, and intended outputs [3].

As an integrated package of services, the intervention relies on a host of elements to ensure programmatic success. Generally, CHWs should be adequately supplied with quality commodities; receive comprehensive training, supervision, and oversight; be embedded in an environment of enabling policy, supervision, and support; and treat communities that are sufficiently knowledgeable and mobilized, among other requisites [4-7]. Although some of these aspects have been explored in isolated detail, the extent to which these act upon each other as an ecology of interdependent elements and how this influences the outcomes of the program is not completely understood. Moreover, the magnitude of their impact on effective coverage and their interactions within the health system at large are not well known; therefore, a more profound exploration of these intervention dynamics is warranted.

Because of the intricate relationships of these elements and the various ways in which different contexts shape their impact, we require an approach that elevates these properties from program isolates acting in silos to interconnected determinants behaving in a complex system. We therefore apply a systems thinking lens to our assessment of the iCCM intervention. Systems thinking is an approach to scientific inquiry that emphasizes the interrelated nature of the composite parts of a system; how these operate within a specific setting; and how these integrate, relate to, and are embedded within the surrounding environment [8].

This protocol builds upon existing systems thinking theory and a methodological approach to assess complexity in community health interventions [9]. This study protocol deals specifically with steps 2, 3, and 4 of the systems approach, namely process mapping, quantitative analysis, and qualitative analysis. In this paper, we outline the specific study design and preliminary results from the data collection process.

### Study Aim

The overall aim of this study is to identify gaps and bottlenecks within critical areas and processes of 4 iCCM programs in Africa, assess factors that are attributable to these, and analyze determinants of programmatic success. Specifically, we are interested in investigating how program design, provider demographics, intervention context, service delivery practices, care recipient experiences and support, and other key health systems areas influence, drive, or are otherwise associated with program outputs of effective coverage. Effective coverage is a metric designed to evaluate the health gain that is experienced by a population, given a certain set of conditions of the health service delivery model [10].

### Study Design

We applied a mixed methods concurrent triangulation design with an intersecting systems theoretical lens [9-12]. Data collection occurs in five discrete tranches:

- Tranche 1: surveys conducted with service providers and recipients of the iCCM intervention (quantitative)
- Tranche 2: focus group discussions (FGDs) conducted with service providers and recipients of the iCCM intervention (qualitative)
- Tranche 3: key informant interviews with program managers, ministry of health (MoH) officers, and other stakeholders at national and district level involved in iCCM activities (qualitative)
- Tranche 4: routine data on cases, service delivery, population and demographics, commodity stocks, reporting, and supervision (quantitative)
- Tranche 5: a policy and document review

**Figure 2:**
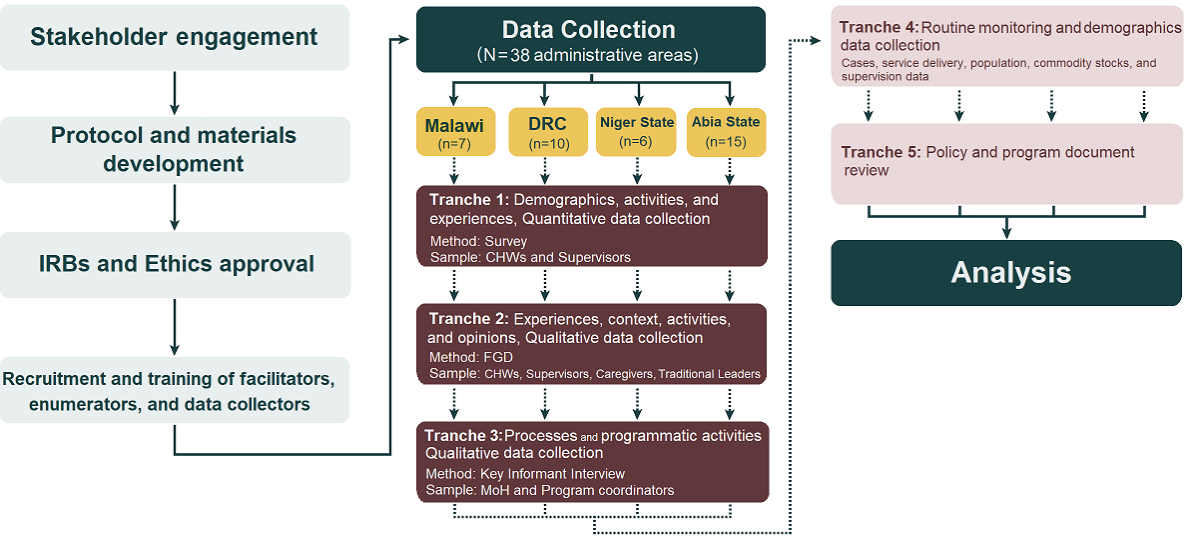
Schematic representation of the research process. CHW: community health worker; DRC: Democratic Republic of the Congo; FGD: focus group discussion; IRB: institutional review board.

An illustration of these tranches is provided in Figure 2.

The study was performed recognizing the existence of different levels of outputs and subsequent outcomes for analysis that will be emergent during the data collection and preliminary analysis period. For example, it is generally accepted that frequent supervision of CHWs is a precondition to successful implementation of community-based programs and thus may serve as an endogenous factor or a process in itself for analysis [13]. Analogously, we may also wish to examine the extent to which the frequency, duration, or other aspect of supervision may affect other activities, elements, or outcomes related to iCCM, such as downstream availability of drug stocks or community ownership of the intervention. The study appreciates that these elements that comprise iCCM do not occur in a vacuum and iteratively influence each other, necessitating a holistic approach to the analysis of the intervention.

## Methods

### Study Setting

The study was implemented in 4 large, geographically distinct territories of 3 sub-Saharan African countries: Malawi, Democratic Republic of the Congo (DRC), and Niger State and Abia State in Nigeria. Within these countries, the iCCM program was administered by the respective state and national ministries of health, with implementation support from local nongovernmental organizations (NGOs) and oversight by the World Health Organization. The program was rolled out across 7 districts in the central and northern regions of Malawi; 10 health zones in the province of Tanganyika in DRC; 6 local government areas (LGAs) in Niger State, Nigeria; and 15 LGAs in Abia State, Nigeria. At the time of the study, iCCM implementation had been ongoing for at least two years. Data were expected to be collected across all these administrative areas where iCCM was implemented.

### Data

The study required both qualitative and quantitative primary and secondary data from different stakeholders and experts within key thematic areas. Primary data were collected from three sources: (1) surveys with CHWs and their supervisors; (2) FGDs with CHWs, supervisors, caregivers and traditional leaders; and (3) key informant interviews with a variety of program partners and country-level stakeholders. An additional survey of caregivers was conducted in Abia State, Nigeria.

We also obtained secondary data to inform the analysis, namely routine monitoring records; household surveys of care-seeking behavior; routine health facility and hospital data (where possible); catchment area demographics, geographical data, and population statistics for communities, health facility areas, and districts; and documentation on program guidelines, manuals, and country child health policies.

### Tool Content and Development

We began with protocol development and drafting of data collection and training materials, specifically survey instruments, FGD guides, and key informant interview templates. We developed 9 survey instruments, 12 FGD guides, and 75 interview guides, varying according to country context. The survey instruments contained between 73 and 137 questions each, the FGD tools contained between 66 and 108 questions per stakeholder group, and the key informant interview guides contained between 25 and 69 questions per informant. These were reviewed for quality and comprehensiveness by funding partners and country team members, which included local academic institutions, country technical representatives, program managers, and MoH members within the country’s child health network. These individuals were identified by the research team through a snowball approach. Each item and country protocol was developed in English and translated into 1 of 10 languages by at least two trained translators recruited either through institutional or local contacts. Translations for the surveys were then subject to a roundtable in-country participatory translation workshop consisting of at least 10 country actors, including program managers or stakeholders, to verify accuracy, relevance, audience appropriateness, and adherence to original question intent.

Surveys and FGDs conducted with CHWs and supervisors focused on their personal profiles, core iCCM processes, inputs, and activities; the caregivers and communities they served; and motivations and opinions. Those conducted with caregivers centered on their knowledge, care-seeking behavior, and adherence, whereas the FGDs conducted with traditional leaders discussed community support, buy-in, and local ownership. The key informant guides collected data on specific iCCM processes, policy, and management of the program, including but not limited to: procurement and distribution of key iCCM commodities; health management information systems; child health policy and program financing processes; human resources; local mobilization; and program coordination, integration, and sustainability at different administrative levels.

### Survey Participants and Eligibility Criteria

The targeted survey participants were CHWs and supervisors. An additional survey with caregivers in Abia State was warranted by the local interest in a statistical measure of the perceived applicability of the intervention in a setting that was geographically considerably smaller and more densely populated than those of the other 3 programs. Eligible CHWs were those who had been trained in iCCM and had participated in the implementation of activities in their communities, whether belonging to a pre-existing health worker cadre or recruited for the specific purpose of this program. Eligible supervisors were health facility attachés who were charged with the supervision of at least one CHW throughout the life of the program. Caregivers in Abia State were eligible participants if they were the primary caretaker of a child aged 2-59 months and resided within the catchment areas of iCCM implementation. Stratified systematic sampling was used for caregiver participant selection. For the CHW and supervisor surveys, bias was mitigated by enumerators cycling through a complete list of all CHWs and supervisors operating in the 38 administrative areas, rather than relying upon local actors to choose participants for the survey.

### Sampling Population

#### Survey

**Figure 3:**
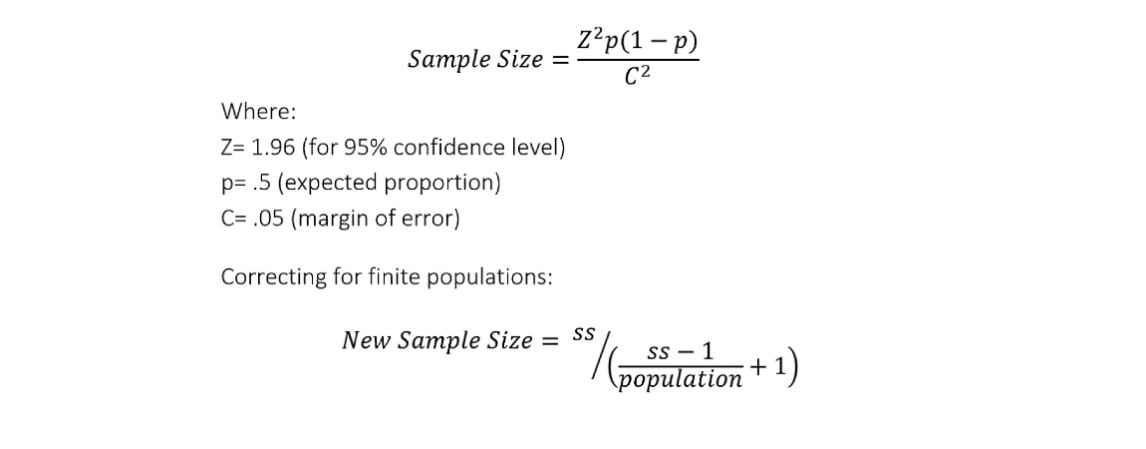
Sample size calculation for surveys.

A single-stage cluster sampling scheme was used for the surveys. All administrative divisions where the iCCM program was implemented in each of the 4 country areas were expected to be sampled. This treated each administrative area as its own independent sampling frame, allowing for full saturation across implementation areas and enabling robust analysis of district-specific effects. The sampling frame for supervisors was at the country area level, with even distribution across all administrative areas. CHW and supervisor population data were sourced from national statistics offices or program records. Survey sample sizes were calculated from these based on probability proportional to size sampling, with aimed representation within a CI of 95% [14]. Figure 3 illustrates the formula for the sample size calculation used to obtain a statistically viable sample of the population within the expected CI. The expected sample sizes and achieved participation for each stakeholder are listed in the *Results* section.

#### FGD Procedures

The FGDs aimed for an average of 8 participants per focus group, which is considered optimal for clarity and representative participation [15]. Saturation of themes is generally expected to occur after 2-3 focus groups; we therefore aimed for 4 FGDs per stakeholder group for the 3 stakeholder groups, where each trine was derived from 1 administrative area [16]. In some country areas, we aimed to hold 1 FGD with traditional leaders to supplement community context.

### Team Recruitment, Enumerator, and Facilitator Training

Enumerator and FGD facilitator guidelines, protocols, and other materials were developed as part of the enumerator training package. We recruited 8-10 survey enumerators per country area according to predetermined criteria with the support of local research teams. These enumerators participated in a round of training resulting in the final selection process; this was followed by a 10-day, 80-hour training course in question content, survey strategy, tablet use, and surveying etiquette. At the end of the course, each enumerator performed 2 test runs of all questions with the survey director from the Swiss Tropical and Public Health Institute and codirectors, usually from the MoH, the supporting NGO, and local university partner. All survey questions were presented with multiple response scenarios, and enumerators were marked based on performance. This also allowed survey supervisors to address any shortcomings in survey structure and design. FGD facilitators were recruited and trained in a similar fashion, where teams consisted of a timekeeper, notetaker, and discussion facilitator.

We adhered to strict compensation guidelines that aim to ensure fair and advantageous remuneration set forth by the International Labor Organization’s fundamental conventions [17-19]. As the recruits were usually young public health professionals, they were also given the opportunity to participate in various capacity-building sessions held as part of the research program.

### Surveys and FGD Rollout

The surveys were piloted over the course of a week, after which final adjustments were made to the questionnaires and enumeration procedures. The official survey data collection lasted on average 2 months per country area. Surveys were carried out with CHWs and their supervisors by trained enumerators by telephone for practicality and cost-effectiveness. CHWs and supervisors were alerted to the study by district health officers at the behest of the investigators and country program managers. Contact information of all CHWs and supervisors was thereafter provided for participant recruitment. Recruitment for the caregiver survey (Abia State) was facilitated by village heads and randomly selected individuals from identified communities. The survey was conducted with electronic data collection using Open Data Kit (ODK), which was programmed on tablets during the tool development stage. Phone credit and headphones were provided to survey participants to carry out the survey. Data were uploaded daily to the secure institutional server. Surveys were conducted in all administrative areas of the iCCM country sites participating in the program.

Establishing contact with CHWs and supervisors proved challenging in some settings as a result of ubiquitous network issues, respondent availability, and general electricity outages. Furthermore, some CHWs had either moved, transferred, resigned, or were deceased, and many had been added who were not updated in the program files. We attempted to control for bias associated with poor network coverage by ensuring a proportional distribution of hard-to-reach (HTR) and easily reached respondents. HTR respondents, meaning those with whom contact was unsuccessful even after 3 attempts, were registered as HTR and were targeted specifically through their supervisor or through the administrative area focal person, who encouraged them to move toward areas of reception or regroup directly at the health facility for the survey. Some enumerators in Abia State traveled to their assigned areas to conduct interviews in person because LGAs were clustered closely together.

The survey interviews typically lasted for 70 minutes each for both survey types. The length of the interview did not deter participants during piloting; therefore, the length was retained. Targeted sample sizes were reached across all country areas, and survey participation was high; 100% of all CHWs and supervisors reached consented to participate. Participants expressed appreciation in having the opportunity to voice their opinions, concerns, and experiences.

For each administrative area, a group of caregivers, supervisors, and CHWs each was chosen to participate in FGDs, averaging 3 FGDs per administrative area and 12 FGDs per country area with these stakeholders. In Niger State and Abia State, additional FGDs were held with local development committees and traditional leaders because of specific interest in these country sites. Participants were recruited with assistance from district health officers and NGO program officers, with a recruitment strategy based on a random sample of participants across communities, health facilities, and districts. The discussions typically lasted between 1½ hours and 2½ hours and up to 3 hours. They were intensive, and hourly breaks were necessary; a stipend, drinks, and food were provided to participants and facilitators. Transport to and from data collection areas was organized either independently, in collaboration with the local ministry or NGO, or through the local research team.

### Quality Control

After the pilots and official launch of the survey, further steps were taken to ensure survey quality. In addition to daily supervision, the principal investigator held triweekly individual meetings with each of the enumerators to discuss results, obtain feedback, and check survey entries. Enumerators were also instructed to obtain correct administrative area, community, and population information for all CHWs and supervisors to revise existing records that were often misaligned with project areas. These data points were corrected in a master registry on institutional servers. Each survey that was conducted was checked against its original entry in the master database, where any errors were rectified. In addition, various roundtable discussions were held and recorded among all enumerators to review emerging themes, outlier anecdotes, concerns, shortcomings, or administrative tasks. Enumerators were also required to take notes and report on specific questions, which were shared with the survey director and recorded in the master file as auxiliary information.

### Ethics Approval and Considerations

This study was approved in 2016 by the institutional review boards of Malawi (NHSRC 16/6/1610), DRC (ESP/CE/046/2016), and Nigeria (NHREC 01012007- 15122016), as well as the Ethics Commission of Northwest and Central Switzerland (EKNZ REQ-2016-00478). All subjects provided informed consent before participating in interviews, discussions, or surveys. All individual data used were anonymized upon collection or extraction; routine monitoring data used existed within a deidentified format. All iCCM programs operated under the supervision of each country’s respective MoH and aligned with national standards of care.

## Results

### Overview

Data collection was undertaken from 2016 to 2017. The following sections outline the experience of preliminary data collection and cleaning in detail. Results and analysis of the implementation of this protocol from each country are in preparation.

### Surveys and FGDs

We successfully collected survey data from CHWs and supervisors across all 38 administrative divisions where iCCM was implemented within the 4 country sites. A total of 3836 surveys were completed. FGDs were conducted in 3-5 administrative areas of each country site resulting in 45 FGDs comprising 379 participants. Statistics on survey and FGD implementation are provided in Table 1.

**Table 1:** Data collection for surveys and focus group discussions (FGDs).

Country site	Administrative area^a^	Survey participants, N=3836							FGD participants, N=378 (45 groups)								
		CHW^b^		Supervisor		Caregiver		CHW				Supervisor	Caregiver		Traditional leader		
**Malawi**																	
	Dedza^c^	118			32	N/A^d^		8		8			10			N/A	
	Lilongwe	128			42	N/A		—^e^		—			—			N/A	
	Mzimba North^c^	81			20	N/A		8		8			9			N/A	
	Nkhatabay^c^	29			42	N/A		8		8			8			N/A	
	Ntcheu^c^	94			8	N/A		8		8			9			N/A	
	Ntchisi	88			8	N/A		—		—			—			N/A	
	Rumphi	67			11	N/A		—		—			—			N/A	
Total			605		163		N/A		32		32			36			N/A
Sample size, 95% CI			305^f^, 598^g^		139^f^		—		—		—			—			—
**Democratic Republic of the Congo**																	
	Ankoro	7			9	N/A		—		—			—			N/A	
	Kabalo	5			14	N/A		—		—			—			N/A	
	Kalemie^c^	75			13	N/A		7		7			12			N/A	
	Kansimba^c^	21			13	N/A		8		5			9			N/A	
	Kongolo	48			17	N/A		—		—			—			N/A	
	Manono	12			10	N/A		—		—			—			N/A	
	Mbulula	8			12	N/A		—		—			—			N/A	
	Moba^c^	41			11	N/A		8		9			10			N/A	
	Nyemba^c^	53			11	N/A		8		8			10			N/A	
	Nyunzu	12			7	N/A		—		—			—			N/A	
Total			282		117		N/A		31		29			41			N/A
Sample size, 95% CI			281^f^		114^f^		—		—		—			—			—
**Niger State, Nigeria**																	
	Edati	89			15	N/A		—		—			—			—	
	Lapai^c^	112			25	N/A		8		8			9			—	
	Mariga	176			26	N/A		—		—			—			—	
	Paikoro	84			15	N/A		—		—			—			—	
	Rafi^c^	141			26	N/A		8		8			9			—	
	Rijau^c^	233			23	N/A		8		8			8			9	
Total			835		130		N/A		24		24			26			9
Sample size, 95% CI			298^f^, 813^g^		117^f^		—		—		—			—			—
**Abia State, Nigeria**																	
	Arochukwu	41			12	—		—		—			—			—	
	Bende^c^	101			16	—		8		8			9			—	
	Ikwuano^c^	109			15	—		8		8			10			—	
	Isialangwa North	24			3	—		—		—			—			—	
	Isialangwa South	99			11	—		—		—			—			—	
	Isuikwuato	54			10	—		—		—			—			—	
	Obingwa^c^	118			14	—		—		—			—			9	
	Ohafia	101			13	—		—		—			—			—	
	Osisioma^c^	105			16	—		8		8			9			—	
	Ugwanagbo	3			1	—		—		—			—			—	
	Ukwa East^c^	12			3	—		—		—			9			—	
	Ukwa West	10			1	—		—		—			—			—	
	Umuahia North	39			11	—		—		—			—			—	
	Umuahia South	40			7	—		—		—			—			—	
	Umunneochi	70			5	—		—		—			—			—	
Total			926		138		640		24		24			37			9
Sample size, 95% CI			294^f^, 892^g^		112^f^		385^f^		—		—			v			—

^a^Administrative areas maintain different nomenclature across countries; accordingly, these subdivisions are *districts* in Malawi, *zones* in Democratic Republic of the Congo, and *local government areas* in Nigeria.

^b^CHW: community health worker.

^c^Administrative areas where FGDs were also conducted.

^d^N/A: not applicable.

^e^Focus group discussions were only conducted in 3 to 4 administrative areas per country site. Caregiver surveys were only collected in Abia State with a representative sample distribution computed at state level.

^f^Minimum sample sizes displayed are national or state level (according to territory distinction); achieved across all territories.

^g^Minimum sample sizes displayed are administrative area level (district, local government area, or zone); specific to CHWs, achieved for Malawi, Niger State, and Abia State.

### Key Informant Interviews

We conducted 120 key informant interviews with a variety of actors fulfilling different roles related to iCCM, including district and zonal government stakeholders; national ministry representatives; national program managers, coordinators, and NGO officers; data managers, pharmacists, and central medical stores affiliates; and field implementers. Program managers and local teams assisted in identifying and requesting participation of key informants. Some follow-up key informant interviews were necessary because of scheduling conflicts. A list of these is provided in supplementary Tables S1-S4 (Multimedia Appendix 1).

### Routine Monitoring, Demographic and Geographic Data, and Document Review

Routine monitoring data were extracted from independent databases held by the iCCM implementing agent (NGO) or the MoH. Country-owned data on care-seeking behavior before and after program implementation were also provided. Permission was requested to access the country’s national health system or to obtain all records and data used, which were usually submitted in digitized format. Country demographic data were obtained from national statistics offices directly or from programmatic registers. These entities also provided access to policy and program documentation.

### Analysis

Statistical data were extracted, cleaned, coded, and analyzed using Stata software (version 15.0; StataCorp LLC). We used nonparametric tests, specifically the Fisher exact test, to assess change and magnitude of effective coverage at critical areas of the iCCM program. Bivariate and multivariate logistic and linear regression analyses were performed to assess factors associated with these critical areas, as well as other emergent programmatic factors. Structural equation modeling was used to measure relationships between variables and latent constructs. Comparative descriptive analyses on routine monitoring data and forms were also performed to evaluate data strength, systems performance, and health provider activities.

Interviews with key informants and FGDs were facilitated, recorded, transcribed, and translated by trained personnel. Transcripts were coded for emergent themes and analyzed according to the Framework Method [20]. Process mapping of key iCCM processes and their bottlenecks was performed using Bizagi software. Causal loop diagrams were used to explore and illustrate programmatic relationships. Full details of the analysis plan have been published separately [8].

## Discussion

### Strengths and Limitations

This systems evaluation protocol benefits from design elements that give ample consideration to the complexity inherent to health systems and programs in low- and middle-income countries. It steps outside the collection of isolated indicators or siloed phenomena to allow exploration of relationships among tangible and intangible facets of the iCCM intervention. As it concentrates not only on program performance, but also on systems performance, it inherently takes into account appropriateness, applicability, and sustainability of the intervention, enabling simultaneously a focused study and an extensive evaluation.

The study protocol includes certain limitations. Data collection of this order is time consuming and can require considerable human resources in both fieldwork and analyses. In addition, because systems studies are necessarily large in scope, parsing apart emergent health systems themes for evaluation can be challenging, while introducing a data burden to the research plan. Potential solutions to address these issues could be using a 2-stage cluster sampling method and samples representative of the national or state level as opposed to administrative areas, a lower threshold for qualitative data saturation (ie, fewer FGDs per key actor and a smaller sample of administrative areas), a narrower focus on specific systems thematic areas, condensed surveys and discussion guides, and remote interviews for those key informants with stable internet access.

### Conclusions

Our proposed study, which capitalizes on the application of the same child health program at wide scale in different contexts, has the potential to advance our understanding of how iCCM programs and other community-based health interventions can operate more effectively and sustainably, what unintended consequences might emerge from their application, and what factors drive the key outputs and impacts of the program. These are essential to achieving Sustainable Development Goal 3.2 of reducing child mortality.

## Appendix

Multimedia Appendix 1 Supplementary Tables S1-S4: Key informant interviews conducted in Malawi, Democratic Republic of the Congo, and Nigeria.

## Abbreviations

CHW: community health worker
DRC: Democratic Republic of the Congo
FGD: focus group discussion
HTR: hard-to-reach
iCCM: integrated community case management
LGA: local government area
MoH: ministry of health
NGO: nongovernmental organization
ODK: Open Data Kit
