# Supplementary material for: Assessing Determinants of Programmatic Performance of Community Management of Malaria, Pneumonia, and Diarrhea in Children in Africa: Protocol and Data Collection for a Mixed Methods Evaluation of Integrated Community Case Management
Source: JMIR Res Protoc. 2022 Mar 14;11(3):e33076. doi: 10.2196/33076 (PMC8961344; doi:10.2196/33076)
Supplement: Multimedia Appendix 1 [file resprot_v11i3e33076_app1.pdf]

Supplementary Material

Table S1. Key informant interviews conducted in Niger State, Nigeria.

Level	Institution	Thematic Area	Key Informant Position (n=34)
**Federal**	Federal Ministry of Health	Policy and governance; finance; supply chain and national logistics; HMIS[b] and data; Supply chain and logistics	Federal iCCM[a] focal person<br>National supply chain officer<br>National HMIS officer<br>National bids and procurement
**NGO-National**	Malaria Consortium	M&E[c] and commodities; program management;	NGO[d] iCCM program director
		Supply chain and logistics	NGO logistics officer
**State**	State Ministry of Health	Policy and governance; finance	High Commissioner<br>Executive State Primary Health Care Director<br>State Director Public Health
		Budget and finance; statistics	Director of Planning, Research and Statistics
		Policy and governance; finance	iCCM coordinator
		HMIS and statistics	HMIS officer<br>State M&E officer
		Service delivery; malaria and policy	Malaria program coordinator
		Supply chain and logistics	State logistic officer
		Service delivery; clinical audits; Refresher training; supervision	State service delivery officer
		Community and social mobilization	Demand creation officer
	SPHCDA[e]	Policy and sustainability	Sustainability group leader
		IMCI and iCCM policy	IMCI[f] coordinator
**NGO-State**	Malaria Consortium	Policy and governance; finance	Program coordinator
		HR[g]; service delivery; recruitment, training, supervision	NGO Service delivery officer
		Social mobilization	Demand creation officer
		Supply chain and logistics	NGO logistic officer
		Budget and finance	Finance officer
		HMIS	Data entry officer
**LGA**	Lapai LGA[h]	Governance; financing;	Lapai Director of Health
		Supply chain; HR	Lapai iCCM focal person
		HMIS	Lapai M&E officer
	Rijau LGA	Governance and financing	Rijau Director of Health
		Supply chain; HR	Rijau iCCM focal person
		HMIS	Rijau M&E Officer
	Rafi LGA	Supply chain; HR	Rafi iCCM focal person
		HMIS	Rafi M&E Officer
	Edati LGA	Supply chain; HR	Edati iCCM focal person

[a]iCCM: integrated community case management.
[b]HMIS: health management information systems.
[c]M&E: monitoring and evaluation.
[d]NGO: nongovernmental organization.
[e]SPHCDA: State Primary Health Care Development Agency.
[f]IMCI: integrated management of childhood illnesses.
[g]HR: human resources.
[h]LGA: local government area.

Table S2. Key informant interviews conducted in Malawi.

Level	Institution	Thematic Area	Key Informant Position (n=30)
National	Malawi Ministry of Health	Policy; finance; sustainability	IMCI[a] Coordinator
		HMIS[b]	National HMIS Officer
NGO[c] - National	Save the Children	Policy; finance	NGO Senior Child Health Manager
		Supply chain; HMIS	Supply Chain Focal Person, Team Leader
District	Dedza DHO[d], MoH[e]<br>Ntcheu DHO, MoH<br>Nkhatabay DHO, MoH<br>Mzimba North DHO, MoH	Supervision;service delivery; supply chain; HR[f]; mobilization; management; sustainability	Dedza District Health Officer<br>Ntcheu District Health Officer<br>Nkhatabay District Health Officer<br>Mzimba North District Health Officer
	Dedza DHO, MoH<br>Ntcheu DHO, MoH<br>Nkhatabay DHO, MoH<br>Mzimba North DHO, MoH	Supervision; service delivery; supply chain; HR; mobilization; management; sustainability	Dedza District Environmental Health Officer<br>Ntcheu District Environmental Health Officer<br>Nkhatabay District Environmental Health Officer<br>Mzimba North Principal Environmental Health Officer
	Dedza DHO, MoH<br>Nkhatabay DHO, MoH	Supply chain and HMIS	Dedza District Pharmacist<br>Nkhatabay District Pharmacist
	Ntcheu DHO, MoH<br>Nkhatabay DHO, MoH<br>Mzimba North DHO, MoH	HMIS and M&E[g]	Ntcheu District HMIS in-charge<br>Nkhatabay District HMIS in-charge<br>Mzimba North District HMIS in-charge
	Dedza DHO, MoH<br>Ntcheu DHO, MoH<br>Nkhatabay DHO, MoH<br>Mzimba North DHO, MoH	supervision; service delivery; supply chain; HR; mobilization; management; sustainability	District IMCI Coordinator<br>District IMCI Coordinator<br>District IMCI Coordinator<br>District IMCI Coordinator
	Dedza DHO, MoH<br>Ntcheu DHO, MoH<br>Nkhatabay DHO, MoH<br>Mzimba North DHO, MoH<br>Mzimba North DHO, MoH	Supervision; mobilization; management	ADEHO[h]<br>ADEHO<br>ADEHO<br>ADEHO<br>ADEHO
NGO - District	Save the Children	Supervision; service delivery; supply chain; HR; mobilization	Save District program coordinator<br>Save District program coordinator<br>Save District program coordinator
Community	Chinteche Village	Mobilization and community engagement	Village Chief

[a]IMCI: integrated management of childhood illnesses.
[b]HMIS: health management information systems.
[c]NGO: non-governmental organization
[d]DHO: district health office.
[e]MoH: ministry of health.
[f]HR: human resources.
[g]M&E: monitoring and evaluation.
[h]ADEHO: assistant district environmental health officer.

Table S3. Key informant interviews conducted in Abia State, Nigeria.

Level	Institution	Thematic Area	Position (n=28)
NGO[a]-National	SFH[b] NGO	Supply chain and national logistics	SFH National logistics officers
State	State Ministry of Health	Policy and governance; finance; sustainability	High Commissioner assistant<br>Executive SPHCDA[c]<br>Permanent Secretary
		Budget; finance; statistics	Director of Planning, Research and Statistics
		Supply chain and logistics; sustainability	Pharmacy director
		HMIS[d] and statistics	HMIS officer
	SPHCDA	Policy and governance; finance; sustainability	Executive Secretary SPCHDA
		IMCI[e] and iCCM[f] pollicy; logistics, service delivery; supervision	iCCM Coordinator
		HMIS	Agency M&E[g] officer
		IMCI and iCCM policy	IMCI coordinator
NGO-State	SFH NGO	Policy and governance; finance	Program coordinator
		HR[h]; service delivery; clinical audit; recruitment; training; supervision	NGO consultant and logistics officer
		HMIS and statistics	NGO HMIS officer
		Budgets and finance	Finance officer
LGA	Ikwuano LGA[i]	Governance; financeing; supervision; sustainability	LGA Head of Department
		HMIS	Data entry officer<br>LGA M&E officer
	Bende LGA	Governance; financing	LGA Head of Department
		Supply chain; HR	LGA iCCM focal person
		HMIS	LGA M&E officer
		Governance; financing	LGA chairman
		Social mobilization and community	Social mobilization officer
	Umuahia North LGA	Supply chain and logistics	LGA pharmacist
		HMIS	LGA M&E officer
		Social mobilization and community	Social mobilization officer
	Osisioma LGA	Supply chain; HR	LGA iCCM focal person
	Ohafia LGA	Supervision and policy; data and supplies	Clinical officer

[a]NGO: nongovernmental organization.
[b]SFH: Society for Family Health.
[c]SPHCDA: State Primary Health Care Development Agency.
[d]HMIS: health management information system.
[e]IMCI: integrated management of childhood illnesses.
[f]iCCM: integrated community case management.
[g]M&E: monitoring and evaluation.
[h]HR: human resources.
[i]LGA: local government area.

Table S4. Key informant interviews conducted in Democratic Republic of the Congo.

Level	Institution	Thematic Area	Key Informant Position (n=28)
**National**	National MoH[a]<br>UNICEF[c]	Policy and governance; finance	IMNMCI[b] program coordinator<br>UNICEF iCCM coordinator 1<br>UNICEF iCCM coordinator 2
**Province**	DPS[d], Province	Policy; finance; supply chain; supervision; M&E[e]; mobilization	Encadreur Provincial
		HMIS[f] and M&E	DPS data manager
**NGO-Province**	IRC[g]	Policy and governance; finance	PCIME[h] coordinator IRC
		HMIS and M&E	HMIS manager
**Zone**	Kalemie Zone, BCZ[i]	Supervision; supply chain; HR[j]; HMIS; management; sustainability	Focal person iCCM[k], BCZ<br>Focal person iCCM, BCZ
	Moba Zone, BCZ		Focal person iCCM, BCZ
	Kansimba, BCZ		Focal person iCCM, BCZ
	Nyemba Zone, BCZ		Focal person iCCM, BCZ
	BCZ, Tanganyika Province		Focal person iCCM, BCZ<br>Focal person iCCM, BCZ
	Kalemie Zone, BCZ	Supply chain and logistics	BCZ pharmacist
	Nyemba Zone, BCZ		BCZ pharmacist
	Kansimba Zone, BCZ		BCZ pharmacist
	Moba Zone, BCZ		BCZ pharmacist
	Kalemie Zone, BCZ	HMIS and M&E	Data management officer
	Moba Zone, BCZ		Data management officer
	Kalemie Zone, BCZ	Supervision; supply chain; HR; HMIS; management; sustainability	MCZ[l]
	Nyemba Zone, BCZ		MCZ
	Kansimba Zone, BCZ		MCZ
	Moba Zone, BCZ		MCZ
**NGO-Zone**	Kalemie Zone, IRC<br>Nyemba Zone, IRC<br>Kansimba, IRC<br>Moba Zone, IRC	Program management; stakeholder relations; supervision; supply chain; HR; HMIS; management; sustainability	IRC focal person<br>IRC focal person<br>IRC focal person<br>IRC focal person

[a]MoH: ministry of health.

[b]IMNMCI: integrated management of neonatal, maternal, and child health.

[c]UNICEF: United Nations Children's Fund.

[d]DPS: divisions provinciales da la santé (provincial health divisions).

[e]M&E: monitoring and evaluation.

[f]HMIS: health management information system.

[g]IRC: International Rescue Committee.

[h]PCIME: prise en charge intégrée des maladies de l'enfance (iCCM of childhood illness).

[i]BCZ: bureau central de la zone de santé (health zone central office).

[j]HR: human resources.

[k]iCCM: integrated community case management.

[l]MCZ: médecin(e) chef de zone (area chief physician).
